# *Aspergillus* appendicitis complicating chemotherapy of leukemia: A case report and review of the literature

**DOI:** 10.1016/j.ijscr.2022.107738

**Published:** 2022-10-12

**Authors:** Kyung Uk Jung, Kyoung Won Yoon, In-Gu Do, Donghyoun Lee

**Affiliations:** a Department of Surgery, Kangbuk Samsung Hospital, Sungkyunkwan University School of Medicine, Seoul, South Korea; b Department of Surgery, Chung-Ang University Gwangmyeong Hospital, Chung-Ang University College of Medicine, Gwangmyeong, South Korea; c Department of Pathology, Kangbuk Samsung Hospital, Sungkyunkwan University School of Medicine, Seoul, South Korea; d Department of Surgery, Jeju National University Hospital, Jeju National University School of Medicine, Jeju, South Korea

**Keywords:** Fungal appendicitis, Leukemia, Aspergillosis, Immunocompromised patients

## Abstract

**Introduction:**

The diagnosis of primary Aspergillus appendicitis can be missed or delayed because of its rarity. We report our experience of a case of Aspergillus appendicitis complicating chemotherapy of leukemia.

**Presentation of case:**

A 48-year-old man who was diagnosed with acute myeloid leukemia developed high fever and epigastric pain two weeks after administration of his fourth consolidation chemotherapy. Right lower quadrant tenderness and rebound tenderness were noticed on physical examination, and the abdomen and pelvis computed tomography suggested acute perforated appendicitis with localized peritonitis. Emergency laparoscopy showed an inflamed appendix, which was resected. Pathology reports revealed invasive aspergillosis in the appendix. The patient recovered after high-dose antifungal therapy, although he required prolonged hospitalization.

**Discussion:**

Acute appendicitis is very rarely caused by fungi infection with an overall incidence of up to 1.15 %. Differential diagnosis of fungal appendicitis without pathology report is challenging due to low incidence.

**Conclusion:**

Isolated Aspergillus appendicitis is a rare disease that can progress without appropriate antifungal therapy even after surgical resection of the appendix. Surgeons should pay attention to pathology reports after appendectomy to avoid missing unusual cases, especially in immunocompromised patients.

## Highlights

• Fungal infections principally occur in the immunocompromised.
• Misdiagnosis and incorrect treatment are frequent in this rare disease entity.
• Early diagnosis and prompt surgery with antifungal treatment is best

## 1 Introduction

Invasive fungal infection is a major cause of mortality in immunocompromised patients, such as those who have undergone transplantation or chemotherapy [1]. With the development of transplantation and chemotherapy, the number of immunocompromised patients is increasing and thus invasive fungal infections are also increasing [2]. Over the past two decades, deaths from invasive mycosis have increased by about 320 % from 1557 to 6534 [3]. Gastrointestinal infection due to fungal infection is a rare but fatal disease entity [4]. Recently, invasive mold infection in stem cell transplantation recipients has been increasing [5]. Most Aspergillus infections occur in the form of a variety of respiratory infections. Extra-pulmonary aspergillosis may be present in 25 %–60 % of cases and is almost universally described in the context of disseminated disease [6], [7], [8]. Although there are a few reports of Aspergillus infections in the pulmonary system, aspergillosis located in the appendix is an extremely uncommon manifestation. In this case report, we report an unusual presentation of invasive Aspergillus infection in a 48-year-old man who had received chemotherapy for acute myeloid leukemia. The report has been written in line with SCARE 2020 guidelines [9].

## 2 Case presentation

**Fig. 1:**
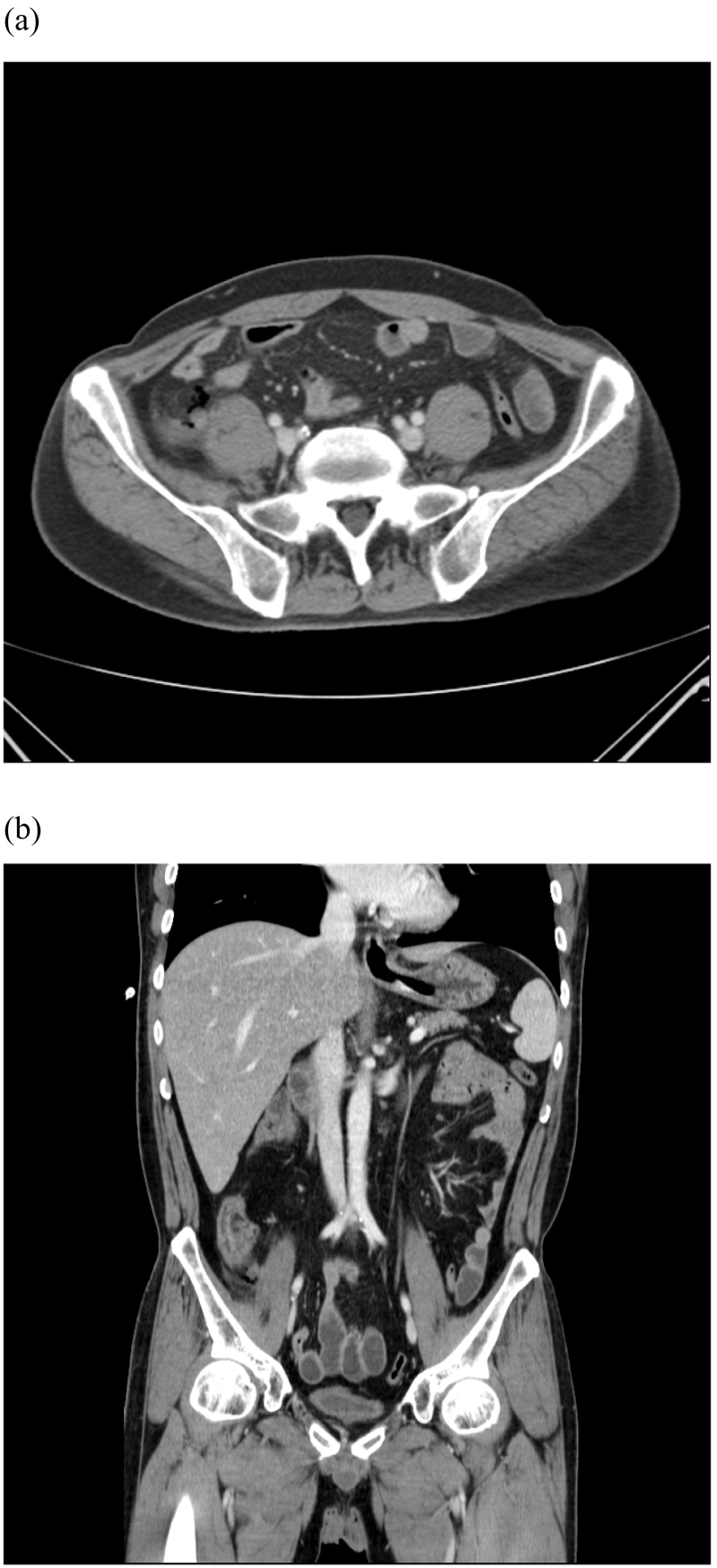
Contrast enhanced axial (a) and coronal (b) CT images at presentation shows perforated appendicitis with air bubble.

**Fig. 2:**
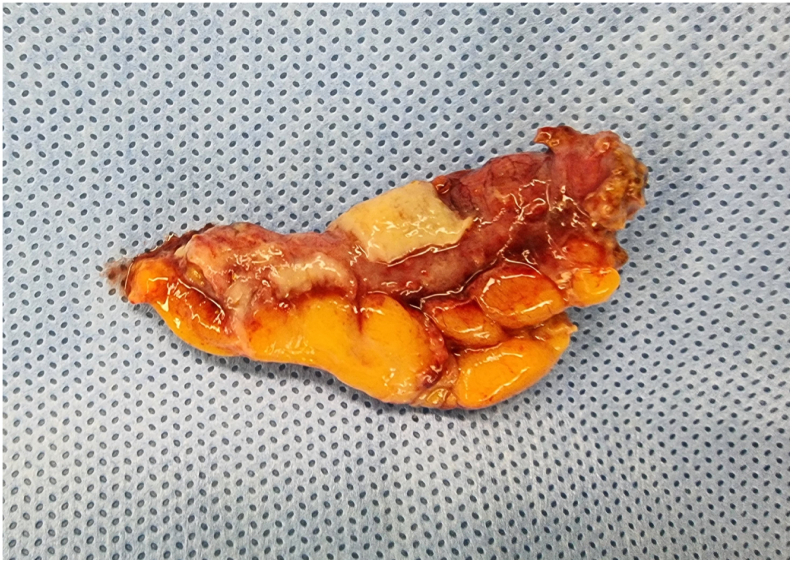
Specimen image demonstrating perforated appendix with white purulent exudate on the serosal surface.

**Fig. 3:**
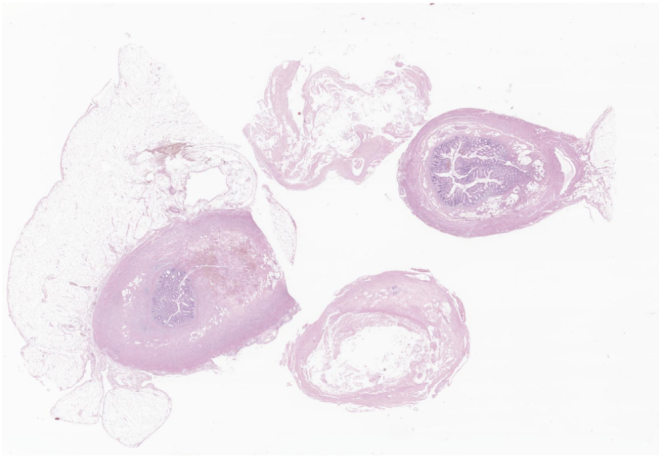
Cross section of resected appendix shows a necrotic and thickened wall due to a severe mixed inflammatory infiltrate.

**Fig. 4:**
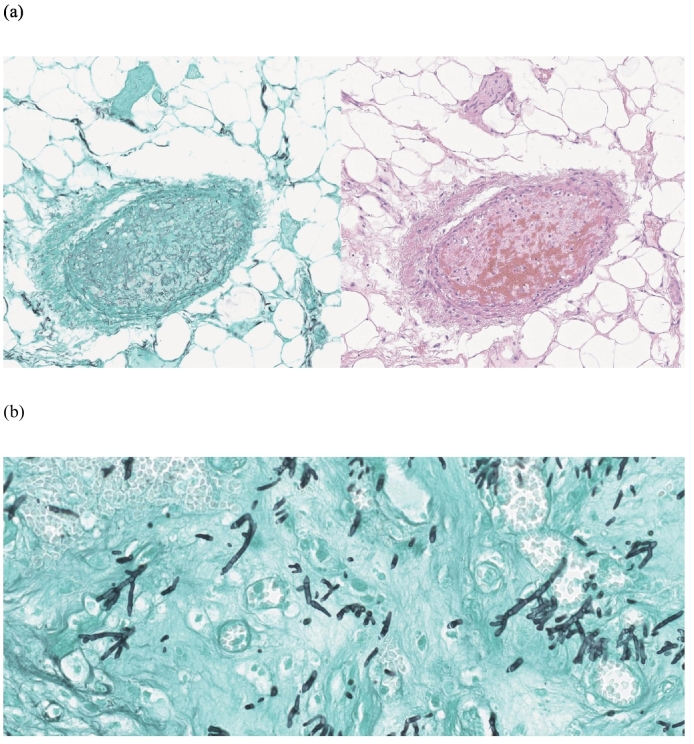
Gömöri methenamine silver stain on left, hematoxylin-eosin stain on right highlighting numerous Aspergillus hyphae (a). Higher magnification (40×) showing abundant septation and acute-angle branching fungal hyphae and numerous spores (b).

A 48-year-old male patient presented following two days of fever and epigastric pain and was admitted to our department. His past medical history was significant for acute myeloid leukemia with a normal karyotype, 46, XY [20], and a 30 % bone marrow blast infiltration, for which he was started on cytarabine plus high-dose daunorubicin for three cycles. He had a high fever (39.3 °C), although blood pressure (127/89 mm Hg) and pulse rate (62 beats/min) were normal. The patient complained of severe epigastric pain, nausea, and persistent diarrhea. Physical examination showed markedly tender and positive rebound tenderness in the right lower abdomen. Laboratory tests showed Hb 6.2 g/dL, white blood cells 0.10 × 10^3^/L (absolute neutrophil count 6/L), platelets 17 × 10^3^/L, and C-reactive protein (CRP) 53.32 mg/dL. Vancomycin was parenterally administered as empiric therapy. Thoracic and abdominal x-rays showed no specific findings. Abdominal computerized tomography showed localized air bubbles in the peri-cecal and peri-appendiceal area with mild perilesional mesenteric infiltration (Fig. 1). These findings were consistent with acute appendicitis, and the patient underwent emergency laparoscopic appendectomy. Laparoscopic findings showed a perforated appendix with focal abscess (Fig. 2). A Jackson–Pratt drain was inserted following appendectomy. Abdominal pain and CRP improved slightly compared with before surgery, but fever (38.8 °C), and mild pain persisted. The pathology report, established on the seventh postoperative day, described an acute perforated appendicitis caused by invasive aspergillosis (Fig. 3). A Gömöri methenamine silver stain revealed many fungal hyphae in the appendiceal lumen and mesoappendix (Fig. 4). While receiving intravenous doripenem and voriconazole. The patient's symptoms gradually resolved by five days without postoperative complications. He was discharged on the eighteenth postoperative day in good general condition and continued antifungal therapy and hematological follow-up. Chemotherapy was resumed without further complications.

## 3 Discussion

Appendicitis is one of the most common conditions that require surgical treatment. In North America, the incidence of appendicitis is 100 per 100,000 person-years with nearly 400,000 diagnoses in 2015 [10]. Recently, in South Korea, the overall incidence of appendicitis was reported as 222.7 per 100,000 population per year [11]. Enteric bacteria are the most common organisms associated with appendicitis. The most common aerobic isolates in appendicitis were *Escherichia coli* and streptococci [12]. In addition, appendicitis may also occur by a parasite such as schistosomiasis, viruses such as measles virus, cytomegalo virus, and Epstein–Barr virus, and fungi. Therefore, it is important to confirm pathology in non-immunocompromised patients who undergo appendectomy.

Recent studies reported that fungal appendicitis occurred in 1.15 % of cases. Thus, a relatively small number of series and single case reports have been published for fungal appendicitis, especially in immunocompromised patients [13], [14]. As a result of the recent increasing use of cytotoxic agents in transplantation and chemotherapy for leukemia, several studies have shown a rising prevalence worldwide of invasive aspergillosis upon autopsy over recent decades [15], [16]. According to a recent study of 1043 hematologic malignancy patients who underwent chemotherapy, about 31 % of those with invasive Aspergillus infection involved the gastrointestinal tract [17]. Specifically, 11.2 % of the invasive Aspergillus infections involved the lower gastrointestinal tract and only 1.3 % caused appendicitis [14], [17]. Fungal appendicitis, in particular, is rare and only a few cases have been reported.

It is known that pulmonary infection, the most common type of Aspergillus infection, is caused by the inhalation of conidia (asexual spore) produced by Aspergillus and dispersed into the air [18]. Most gastrointestinal aspergillosis usually occurs via dissemination from a primary pulmonary infection. Therefore, primary gastrointestinal aspergillosis including appendicitis alone is rarely seen without pulmonary infection [19], [20]. The pathogenesis of Aspergillus appendicitis is unclear. Based on existing studies, Aspergillus species may invade through the gastrointestinal tract after disruption of the intestinal mucosal barrier caused by chemotherapy, or they may disseminate from the lung to the gastrointestinal tract through hematogenous spread [21], [22].

The clinical features of Aspergillus appendicitis that distinguish this from bacterial appendicitis are not clear and will most likely resemble those of bacterial appendicitis. Symptoms of Aspergillus appendicitis are diverse, ranging from non-specific vague abdominal pain, fever, and nausea to tenderness or rebound tenderness in the right lower quadrant. Despite recent advances in radiologic technology, there is no other suitable method for diagnosis of Aspergillus appendicitis other than histopathologic study. It is difficult to diagnose aspergillosis using blood culture. For immunocompromised patients at risk of fungal infection, abdominal computed tomography, which is the investigation of choice in appendicitis, should be performed without delay if clinical manifestations of appendicitis are suspected.

Since it was first performed in 1981, laparoscopic appendectomy has been the gold standard treatment for appendicitis [23]. Although it is rare to find large-scale research except for few case reports on surgery for fungal appendicitis, laparoscopic appendectomy is considered as the preferred treatment so that fungal dissemination can be avoided. In that case, it can be best to follow a laparoscopic approach due to minimal invasiveness. In the case of the particular patient reported here, we performed reduced port appendectomy.

In addition to surgical intervention, if histopathologic confirmation is obtained after surgery, a systemic antifungal agent must be administered to prevent disseminated fungal infection. Fungal appendicitis alone is exceedingly rare but disseminated aspergillosis with pulmonary infection can be fatal even in patients who are not immunocompromised [24]. Amphotericin B and triazole antifungal agents including voriconazole, posaconazole, itraconazole, and fluconazole are commonly used drugs for invasive Aspergillus. A recent study suggests that initial therapy with voriconazole leads to better responses, improved survival, and fewer severe side effects than the standard approach of initial therapy with amphotericin B in invasive Aspergillus infection [25]. The dosage for patients aged ≥12 is 6 mg/kg twice daily on day 1 followed by 4 mg/kg twice daily for the remainder of treatment. [26].

## 4 Conclusion

In conclusion, Aspergillus appendicitis is difficult to diagnose preoperatively, and surgery is required in most cases. A laparoscopic approach could be an effective treatment. Surgeons should pay attention to histopathologic confirmation after appendectomy in order not to miss unusual cases, especially in immunocompromised patients.

## Provenance and peer review

Not commissioned, externally peer-reviewed.

## Consent

Written informed consent was obtained from the patients for publication of this case report and any accompanying images. A copy of the written consent is available for review by the Editor-in-Chief of this journal.

## Ethical approval

No approval required.

## Funding

There are no sponsors or special funding for writing or publication of this case report.

## Guarantor

Donghyoun Lee.

## Research registration number

1. Name of the registry: –
2. Unique identifying number or registration ID: –
3. Hyperlink to your specific registration (must be publicly accessible and will be checked): –.

## CRediT authorship contribution statement

Kyoung Won Yoon and Kyung Uk Jung equally contributed to manuscript writing and editing; In-Gu Do contributed to pathologic analysis; Donghyoun Lee generally supervised whole process; all authors have read and approved the final manuscript.

## Declaration of competing interest

The authors have no conflicts of interests.
